# Isolated urethral tuberculosis in a middle-aged man: a case report

**DOI:** 10.1186/1752-1947-7-97

**Published:** 2013-04-08

**Authors:** Ahmed-Amine Bouchikhi, Driss Amiroune, Mohammed Fadl Tazi, Soufiane Mellas, Jalal Eddin Elammari, Mohammed Jamal El Fassi, Adelhak Khallouk, My Hassan Farih

**Affiliations:** 1 Urology Department, University Hospital of Fez, Rue Zag, Rce Andalous III, Quarier Al-Wafe, Fès, 30070, Morocco

**Keywords:** Tuberculosis, Male urethra, Urethral stricture, Fistula

## Abstract

**Introduction:**

Urogenital tuberculosis is a frequent disease in endemic countries. It is characterized by clinical polymorphism. The isolated urethral form is exceptional, even in countries with endemic tuberculosis. We present a rare case of urogenital tuberculosis in a man revealed by urethral narrowing and multiple urethro-scrotal fistulas.

**Case presentation:**

The patient, a Moroccan man, was 40 years old. He visited our hospital for a urology consultation and presented with dysuria, purulent discharge and a meatic penoscrotal fistula. A retrograde and voiding urethrocystography was performed and revealed an extended narrowing of the whole anterior urethra associated with multiple fistulous portions toward the scrotum and perineum. At this stage, we reached a diagnosis of nonspecific sclero-inflammatory urethral stricture with complicating fistulas. We decided to perform a urethroplasty enlargement to clear the narrowing urethral sinus tracts. The evolution was marked by delayed wound healing associated with the persistence of fistulas extending into the corpus cavernosum with purulent discharge. It was at this point in the treatment that we suspected tuberculosis. Multiple biopsies were then performed on the periurethral tissue and fistula tracts. The histological examination confirmed urethral tuberculosis and showed the presence of giant cell epithelial lesions with caseous necrosis characteristic of tuberculosis. The treatment for tuberculosis was immediately established and the evolution was marked by a localized, rapid and significant improvement. A second-stage urethroplasty was scheduled for two months after the start of his antituberculous treatment.

**Conclusions:**

Urogenital tuberculosis is common, but isolated urethral involvement is very rare even in countries with endemic tuberculosis. We urge practitioners, and especially urologists, to consider the disease in their investigation whenever given clinical signs are declared.

## Introduction

Urogenital tuberculosis (TB) is a frequent disease in endemic countries. It is characterized by clinical polymorphism. The isolated urethral form is exceptional, xeven in countries with endemic tuberculosis. We present a rare case of urogenital tuberculosis involving an isolated urethral form in a man revealed by urethral narrowing and multiple urethro-scrotal fistulas. We discuss the diagnosis and treatment considerations and review the literature. Finally, we recommend that practitioners consider the disease in endemic countries based on clinical features.

## Case presentation

**Figure 1:**
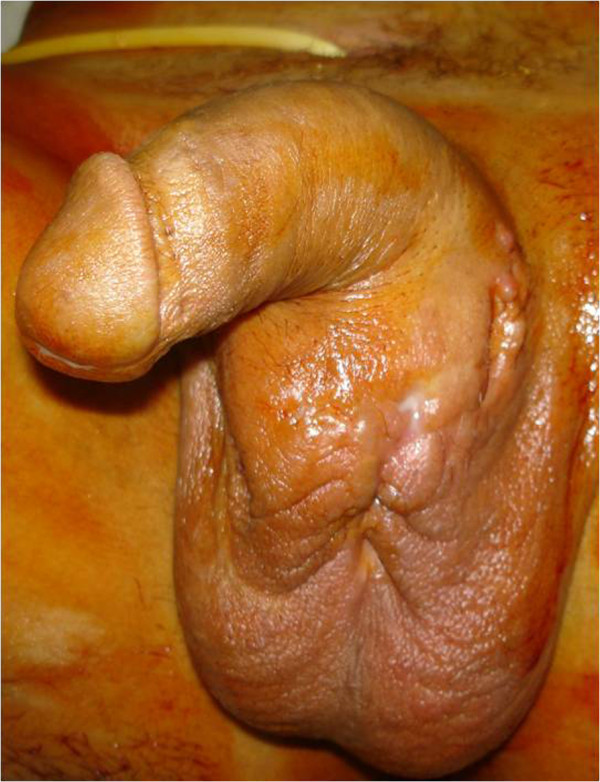
Multiple fistulated penoscrotal orifices with pus excretion.

The patient was a 40-year-old Moroccan man who had been vaccinated with bacillus Calmette–Guérin (BCG). He did not have any signs of previous TB infection or a history of sexually transmitted infections. The patient visited our hospital for a urology consultation and presented with dysuria, purulent discharge and a meatic penoscrotal fistula that had developed over a one-year period. The physical examination at admission found nodular lesions, urethral induration on the urethral path extending laterally to the cavernous bodies with multiple fistulas, and penoscrotal pus excretion (Figure 1). The external genitalia examination, testis, epididymis and ductus deferens, were without remarkable signs. His prostate volume was normal with a soft consistency. His prostate volume was normal with a soft consistency.

**Figure 2:**
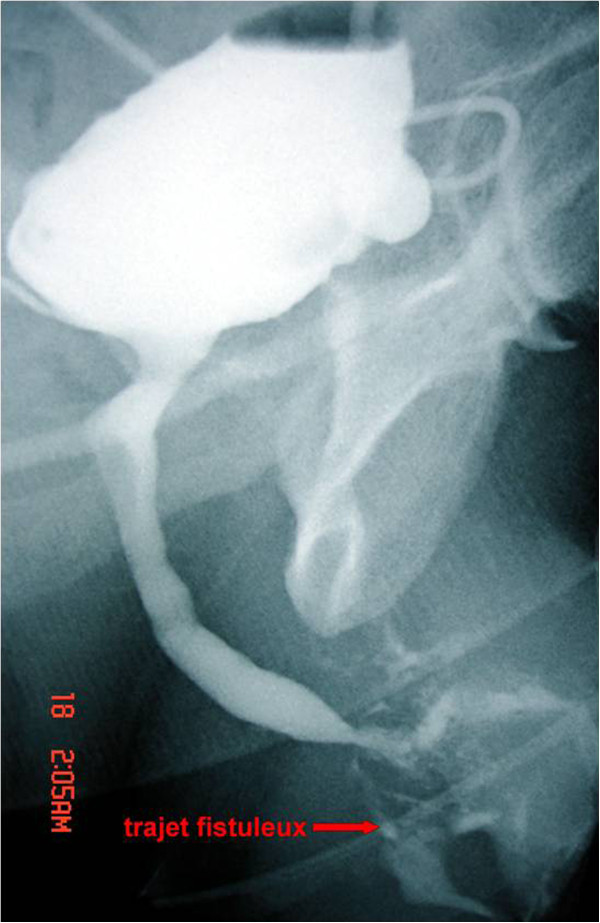
Urinary retrograde and voiding urethrocystography shows an anterior narrowing of the urethra with infiltration of the contrast agent in the sinus trajectory.

**Figure 3:**
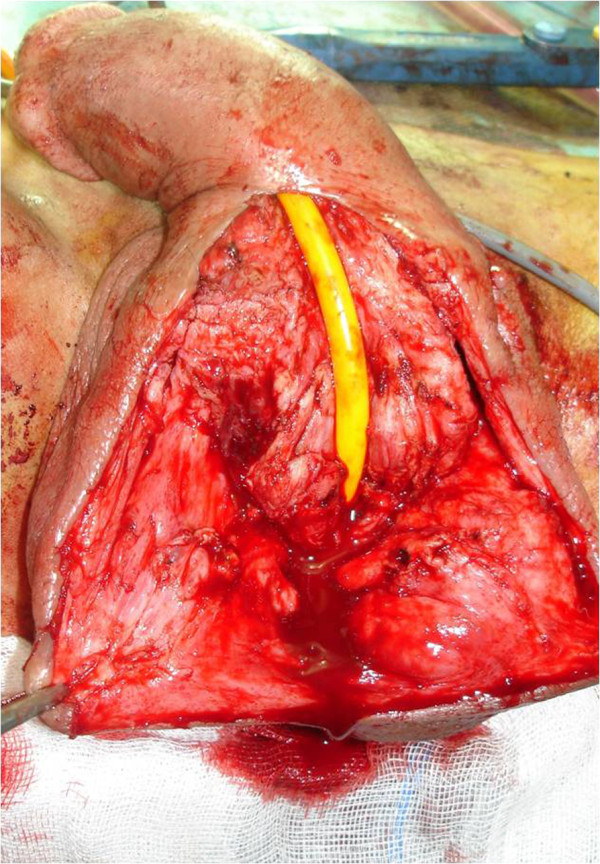
Peri-operative picture shows the flattening of the urethra and fistula trajectory.

The initial biological assessment revealed an inflammatory syndrome corresponding to a sedimentation speed of 80 associated with a urinary tract infection with *Escherichia coli* susceptible to fluoroquinolones. First, the patient received antibiotherapy based on ciprofloxacin and benefited from urinary drainage by suprapubic catheter. A retrograde and voiding urethrocystography (UCG) was then performed and revealed an extended narrowing of the whole anterior urethra associated with multiple fistulous portions toward the scrotum and perineum. The bladder control showed a bilateral secondary vesico-ureteric reflux (Figure 2). At this stage, we reached a diagnosis of nonspecific sclero-inflammatory urethral stricture with complicating fistulas. After six weeks of urinary drainage and antibiotherapy, we decided to perform a urethroplasty enlargement to clear the narrowing urethral sinus tracts (Figure 3).

The evolution was marked by delayed wound healing associated with the persistence of fistulas extending into the corpus cavernosum with purulent discharge. It was at this point of the treatment that we suspected TB and we carried out a biological assessment in this regard. His test result for Koch bacillus (BK) in the urine was negative. His tuberculin assessment result was positive. Multiple biopsies were then performed on the periurethral tissue and fistula tracts.

The histological examination confirmed urethral TB and showed the presence of giant cell epithelial lesions with caseous necrosis characteristic of TB. The treatment for TB was immediately established and marked by a localized, rapid and significant improvement. A second-stage urethroplasty was scheduled for two months after the start of his anti-TB treatment.

## Discussion

Urogenital localization is the second most common localization of extrapulmonary TB [1]. It might affect all urogenital tracts, with severe consequences as it may adversely affect renal function through the urological tracts as well as introduce genital damage that would harm the genital and sexual functions including the epididymis, the vas deferens channels, and the ejaculatory ducts, leading to impaired fertility conditions such as excretory azoospermia.

Isolated urethral involvement is very rare. The infection pathway is essentially through a spread from a neighboring contaminated site, which is often the prostate. This explains the frequent association of prostate and urethra affection. However, BK affection of the urethra spongy body through blood is possible [2-4]. It is probable that the stasis of urine infected by KB in the urethra has a major role in developing urethral TB [1]. The isolated urethral form of TB, as was the case with our patient, is rare [5,6].

The clinical profile is often nonspecific and confusing. The urethral affection might occur in the acute form of acute urethritis associated with severe prostate or seminal vesicle involvement in men, or it may evolve into a chronic form of urethral narrowing with a sclero-inflammatory mode that is often extended and associated with unusual multiple fistulas [6,7].

Sometimes, the diagnosis is suspected after an initial full voiding, as was the case with our patient, since fistulas are still persistent despite urinary drainage, and urethra and fistula clearing. The diagnosis is highly suspected in a patient with TB contagion signs, previous TB infection or those known to be active TB carriers. In addition to retrograde and voiding UCG, allowing exploration of the extent and severity of urethral lesions, it is essential to investigate the rest of the urogenital tracts, possibly targeting other localizations such as by renal ultrasonography, intravenous urography, uroscan, and so on.

The diagnosis is confirmed by the biological and/or the anatomopathological assessments. Indeed, the urine culture confirms the diagnosis when it is used to isolate acid-alcohol-resistant bacilli-resistant species (AFB). However, this test is rarely positive. Thus, it is essential in the case of strong suspicion to achieve a culture from the urine sample on a specific medium (Lowenstein), this allows confirmation of the diagnosis and identification of the sensitivity to the anti-KB bacilli. The drawback of a bacilli culture is that it takes eight weeks, which is very long time. The AFB identification in the urine by polymerase chain reaction (PCR) is faster and takes 24 to 48 hours with a sensitivity ranging from 48.5 to 95% and a specificity of 98% [8].

Newer immunoassay assessment techniques based on quantifying interferon gamma production by memory T cells from the patient are still in the evaluation stage.

Anatomopathological examinations of periurethral scar tissue and fistula tracts are very specific to TB when they reveal epithelioid granulomas with giant cell caseous necrosis.

The treatment is based on a poly-antibiotic anti-bacillary regimen lasting at least six months. This medical treatment must be completed six to 12 weeks before any surgical intervention. This allows prevention of the reactivation of latent forms in scar tissue and dissemination of BK in healthier tissue during surgery [6,9]. The remaining treatment of strictures has to be similar to nonspecific forms. It is preferred that expansion is achieved via urethroplasty, considering the prominent fibrosis that is often found in the urethra and other severely affected urethral tissues.

## Conclusions

Urogenital TB is common, but isolated urethral involvement is very rare even in countries with endemic TB. We suggest considering urethral TB before a non-specific urethral stricture in patients living in endemic area of TB. We suggest considering urethral TB before a urethral stricture in patients living in endemic area of TB.

## Consent

Written informed consent was obtained from the patient for publication of this case report and any accompanying images. A copy of the written consent is available for review by the Editor-in-Chief of this journal.

## Competing interests

The authors declare that they have no competing interests.

## Authors’ contributions

AAB was the principal author and major contributor in writing the manuscript. MFT, SM, JEE, AK and DA analyzed and interpreted the patient data and reviewed the literature. MJE, AK and MHF read and corrected the manuscript. All authors read and approved the final manuscript.

## References

[B1] Palaniswamy R Bhandari M Urethral fistulae of tuberculous origin Singapore Med J 1984 25 54 56 6540474

[B2] Symes JM Blandy JP Tuberculous of the male urethra Brit J Urol 1973 54 432 436 10.1111/j.1464-410x.1973.tb12184.x 4199709

[B3] Chambers RM Tuberculous urethral fistula Brit J Urol 1971 43 243 248 5579481 10.1111/j.1464-410x.1971.tb12033.x

[B4] Dje AK Yao AB D’Horpock BFA Tchimou J La tuberculose urogénitale: difficultés diagnostiques Ann Urol (Paris) 2003 37 233 235 10.1016/S0003-4401(03)00091-3 14606308

[B5] Gupta NP Kumar R Reconstructive surgery for the management of genitourinary tuberculosis: a single center experience J Urol 2006 175 2150 2154 10.1016/S0022-5347(06)00310-7 16697825

[B6] Gupta N Mandal AK Singh SK Tuberculosis of the prostate and urethra: a review Indian J Urol 2008 24 388 391 10.4103/0970-1591.42623 19468474 PMC2684345

[B7] McAleer SJ Johnson CW Johnson WD Wein AJ, Kavoussi LR, Novick AC, Partin AW, Peters CA Tuberculosis and parasitic and fungal infections of the genitourinary system Campbell-Walsh Urology 2007 9 Philadelphia: WB Saunders 436 470

[B8] Yazdani Kachoei M Karami A Baradaran S Shirani M Yazdani A Diagnostic value of urine polymerase chain reaction in genitourinary tuberculosis Urology 2009 74 4 S245

[B9] Koutlidis N Fillion A Michel F Tuberculosis urogenital EMC - Urología 2009 41 3 1 12

